# Insight into the Toxicological and Pathophysiological Effects of Moroccan Vipers’ Venom: Assessing the Efficacy of Commercial Antivenom for Neutralization

**DOI:** 10.3390/tropicalmed8060302

**Published:** 2023-05-31

**Authors:** Soukaina Khourcha, Ines Hilal, Iatimad Elbejjaj, Mehdi Karkouri, Amal Safi, Abdelaziz Hmyene, Naoual Oukkache

**Affiliations:** 1 Laboratory of Venoms and Toxins, Pasteur Institute of Morocco, Casablanca 20360, Morocco; drineshilal@gmail.com (I.H.); naoual.oukkache@pasteur.ma (N.O.); 2 Laboratory of Biochemistry, Environment and Food Technology, Faculty of Sciences and Technologies of Mohammedia, Mohammedia 20650, Morocco; 3 Laboratory of Pathological Anatomy, University Hospital Center Ibn Rochd, Casablanca 20360, Morocco

**Keywords:** *Bitis arietans*, *Daboia mauritanica*, *Cerastes cerastes*, toxic activities, physiopathology, antivenom, venom neutralization

## Abstract

Morocco is one of the richest countries in biodiversity in the Mediterranean region, especially in its ophidian fauna. In total, there are eight species of venomous snakes, with seven belonging to the Viperidae family, responsible for 67.2% of severe envenomation cases in the country. *Cerastes cerastes*, *Daboia mauritanica* and *Bitis arietans* are considered among the most venomous vipers whose bites cause high levels of morbidity, disability or mortality. Despite their wide distribution in the kingdom, the incidence of these snakebites remains poorly understood and largely underestimated. Moreover, intraspecific variations in the venom composition significantly affect the effectiveness of antivenoms. Due to the unavailability of locally produced antivenoms, we evaluated the efficacy of *Inoserp-MENA*, the only available antivenom in Morocco, against *C. cerastes*, *D. mauritanica* and *B. arietans.* First, we conducted a comprehensive characterization of these venoms, including an LD_50_ test to examine their toxicity and SDS-PAGE as a technique to analyze the enzymes responsible for biological activities, such as hemorrhagic and edematous activities and myotoxicity, which generate physiopathological effects in the skin, paws and muscles of envenomed mice. Then, we assessed the ability of *Inoserp-MENA* antivenom to neutralize the toxic activities of Moroccan vipers. Our results indicate that the venom of *C. cerastes*, *D. mauritanica* and *B. arietans* are toxic, causing severe alterations such as edema, myotoxicity, myonecrosis and significant hemorrhages with the formation of hemorrhagic foci. *C. cerastes* venom is more dangerous in terms of lethality and hemorrhages, while *B. arietans* venom is more edematous. The effects of *C. cerastes* venom were effectively neutralized, but *Inoserp-MENA* antivenom failed to protect mice against the toxic effects induced by *B. arietans* and *D. mauritanica* venom. The study reveals alarming shortcomings in the effectiveness of the current commercially available antivenom’s dosage and neutralization capabilities, highlighting the urgent need to develop a region-specific viper envenomation therapy.

## 1. Introduction

Nowadays, snakebite envenomation is a major public health problem and is considered a neglected tropical disease according to the World Health Organization (WHO), especially in developing countries [1]. Worldwide, 95% of snake bites (averaging 5 million) occur in rural areas, leading to 125,000 deaths and causing three times more amputations and permanent disabilities [2]. Reports of snake bite mortality and morbidity suggest that Asian countries are listed at the top of the most affected countries, followed by Sub-Saharan Africa [3]. In Africa, snake bites lead to 32,000 deaths per year, with permanent disabilities and sequelae. The most dangerous and medically relevant species of venomous snakes in East, West and Central Africa belong mainly to the genera *Bitis, Echis, Daboia, Cerastes* and *Naja* (WHO, 2010).

In Morocco, snakebite envenomation is a very worrying issue for rural populations and health professionals, and is caused by snakes belonging to two major families: *Elapidae* and *Viperidae* [4]. The latter family is the most incriminated in severe envenomation cases (67.2%) and is mainly represented by vipers, i.e., *Cerastes cerastes*, *Daboia mauritanica* and *Bitis arietans* [5]. According to the WHO, the *puff adder* (*B. arietans*) is one of the most highly venomous snakes, whose bites cause high levels of morbidity, disability or death (i.e., Category 1 species) [6]. Viper envenomation particularly disturbs the circulatory system by altering the hemostasis of the prey/victim, which in turn causes death by uncontrolled bleeding. In addition, it also induces myotoxicity, nephrotoxicity and serious local manifestations, in particular myonecrosis, dermonecrosis, extracellular matrix (ECM) degradation, hemorrhage and edema [7,8]. The physiological effects observed during ophidian envenomation can be attributed to the complex composition of venom, comprising a diverse array of biological substances such as enzymes, toxins and peptides. Proteomic analyses of the venom from *Cerastes cerastes* and *Daboia mauritanica*, as demonstrated in studies by Calvete and collaborators, have provided valuable insights into their proteomic profiles [9]. A proteomic analysis of *Cerastes cerastes* venom revealed the presence of phospholipase A_2_, metalloproteinases and serine proteases among the enzymatic components. Additionally, the venom exhibited toxins including cerastocytin, a disintegrin-like protein and snake venom serine proteinase inhibitors. Similarly, a proteomic analysis of *Daboia mauritanica* venom identified a diverse range of proteins, including metalloproteinases, phospholipases A_2_, serine proteases and C-type lectin-like proteins. Notable toxins in this venom included factor X activator, vascular endothelial growth factor and snake venom serine proteinase inhibitors [10]. However, it is noteworthy that the proteome of the viper *Bitis arietans* collected in Morocco remains unexplored, as mentioned in the original statement. Consequently, the specific composition of its venom and the presence of particular proteins and toxins are yet to be elucidated. These proteomic findings highlight the species-specific variations in the nature, function and effects of the molecules present in snake venom. These biological substances can exert their effects individually or synergistically, thereby inducing a wide range of local and systemic physiopathological changes observed during snake envenomation [8,9].

Antivenoms remain the only specific treatment for snakebite envenomation, but their effectiveness is limited in many regions, making it a major challenge in the global fight against snakebites. According to several studies, selecting the appropriate antivenom depends on identifying the snake species responsible for the bite. Although the clinical symptoms vary significantly, they pose the most significant obstacle in treating snakebite victims, highlighting the need for improved antivenom production to provide effective treatment [11]. Notably, *B. arietans* venom was collected in Morocco and studied for the first time in our work. However, North African *C. cerastes* and *D. mauritanica* venom have been previously studied in several works and showed different levels of clinical toxicity, because although belonging to the same species, these venom have distinct envenomation characteristics. To address this knowledge gap, we aim to conduct a thorough study of the venom from these three vipers, encompassing biochemical, enzymatic, toxicological and biological characterizations. Additionally, we will assess the effectiveness of the only available and marketed antivenom in Morocco, *Inoserp-MENA*, using mice tests recommended by the World Health Organization (WHO) to counteract the effects of the venom WHO [12]. The findings of our study reveal significant variations in venom composition, biochemical effects and toxicity profiles, as well as the low neutralization capacities of commercial antivenom, underscoring the pressing need for the development of a targeted treatment for snakebites in this region.

## 2. Materials and Methods

### 2.1. Venoms and Antivenom

**Figure 1:**
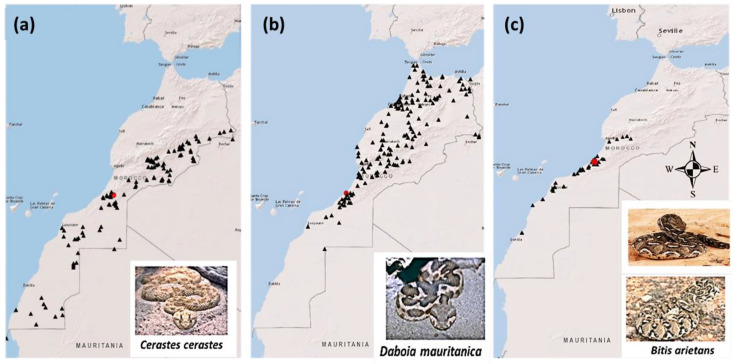
Geographical distribution of *Cerastes cerastes* (**a**), *Daboia mauritanica* (**b**) and *Bitis arietans* (**c**). Each species tends to select habitats with different geographical locations. The geographical distribution of *Cerastes cerastes*, *Daboia mauritanica* and *Bitis arietans* varies across different habitats and locations. (**a**) *Cerastes cerastes* vipers are commonly found in the desert and arid regions of the Sahara, particularly in the eastern and southern parts of Morocco. (**b**) *Daboia mauritanica* is typically found in rocky areas with shrubs and cactus vegetation, predominantly in the northern, western and central regions of Morocco. (**c**) *Bitis arietans* vipers have a specific distribution along the southern coastal region of Morocco [14].

Twenty adult specimens for each viper (*Cerastes cerastes* (*C. cerastes*), *Daboia mauritanica* (*D. mauritanica*) and *Bitis arietans* (*B. arietans*)) were collected from different regions of Morocco with a high risk of snakebite envenomation (Figure 1). The crude venom of the three vipers was extracted from their fangs after manual stimulation of the venom gland, raised at the serpentarium of the Pasteur Institute of Morocco (PIM). The extracted venom was diluted with a small volume of cold water and centrifuged at 13,000 g at 4 °C for 15 min. The supernatants were lyophilized and then frozen at −20 °C until use [13]. The same batches of these venoms were used for each analysis to ensure continuity of the study. *Inoserp-MENA* antivenom is a specific polyvalent immunotherapeutic F(ab’)2 snake antivenom, provided in the lyophilized form and indicated for the treatment of patients presenting clinical signs of snake envenomation by species such as *Bitis arietans, Cerastes cerastes, Echis leucogaster, Macrovipera lebetina obtusa, Naja haje, Naja pallida, Naja nigricollis, Vipera palestinae, Walterinnesia aegyptia* and *Macrovipera deserti*. It was provided by the antipoison control center (CAPM). The batch number of *Inoserp-MENA* antivenom used in this study was 0IT09001, manufactured in September 2020, with an expiration date of September 2023.

### 2.2. Mice and Ethics Clearance

Male Swiss mice weighing 18–22 g were sourced from the animal unit of the Pasteur Institute of Morocco for use in various experiments. The animals were kept under standard temperature conditions (22 ± 2 °C) and a 12 h/12 h photoperiod, with ad libitum access to food and water throughout the study. All the tests and procedures involving animals strictly followed the ethical principles in animal research recommended by the World Health Organization [15]. The Ethics Committee of the Pasteur Institute of Morocco, under agreement number 8.3.A-2015, authorized animal testing on 15 April 2015.

### 2.3. Protein Concentration

The protein content of the three venoms and antivenom was evaluated using the Bradford quantification method [16]. For this task, a six-point calibration curve in the range of 0 to 10 µg/µL was used with a Bovine Serum Albumin BSA solution of 100 ug/mL. The venoms were diluted in the range of 1/1000 to 1/128,000 and 200 µL of Bradford reagent was then added to the samples. After 20 min, the absorbance was measured at 595 nm.

### 2.4. SDS-PAGE

First, the venoms were pretreated in reducing conditions using 5% β-mercaptoethanol. An amount of 20 µg of crude venom was diluted in loading buffer under reducing conditions (b-mercaptoethanol, 2-ME) and loaded on 12% SDS-polyacrylamide gel [17]. The molecular weights were estimated by comparing the venom protein bands with protein markers (14.4–97 kDa) (Bio-Rad, Hercules, CA, USA). Each sample was boiled for 5 min and 120 V was applied for 60 min using a vertical electrophoresis system (Bio-Rad). After separation, the gels were stained with Coomassie blue R-250 for 2 h and rinsed with 40% methanol and 10% acetic acid.

### 2.5. Assessment of the Toxic and Enzymatic Activities of Venoms

A group of Male Swiss mice weighing 20 ± 2 g was used to conduct in vivo experiments to assess the lethality of the venom of three vipers (*C. cerastes*, *D. mauritanica* and *B. arietans*), as well as its ability to induce hemorrhage, edema, myotoxicity and histological damage.

#### 2.5.1. Lethality

The median lethal dose (LD_50_) is defined as the amount of venom that kills 50% of the population of animals injected. Groups of five male Swiss mice were administered different doses of venom, with a final injected volume of 0.5 mL. The mortality percentage was recorded 24 h after intraperitoneal injection. The values of the LD_50_ were calculated through non-linear regression (variable slope) using GraphPad Prism 7.0 software [18] and expressed in µg/mouse and µg of venom per gram of body weight (µg/g).

#### 2.5.2. Hemorrhagic Activity

Hemorrhagic activity was assessed as described by measuring the formation of the hemorrhagic halo in dorsal skin [19]. For each venom, eight groups of four mice received an intradermal injection into the dorsal skin of increasing doses of venom (0.025 to 1 µg/g) diluted in a sterile physiological saline solution. A control group was used for the three venoms and received an injection of 100 µL of a sterile physiological saline solution. The skin was removed after 2 h and the diameters of the hemorrhagic spots were measured on the internal surfaces. The minimum hemorrhagic dose (MHD) was defined as the venom dose that induced a lesion of 10 mm in diameter.

#### 2.5.3. Edema Forming Activity

The minimum edema forming dose (MED) of each venom pool was determined by measuring the amount of venom that induces 30% edema compared to the control. The test was performed according to previous studies [20,21]. Increasing doses of venoms were dissolved in 50 μL of saline dilutions (0.1 to 10 μg/mouse) and were injected into the footpads of the right hind limb (i.pl) of eight groups of four mice. As a negative control, 50 μL of saline solution was injected into the left limb of the mice. The mice were euthanized and their hind limbs were amputated at the ankle joints after 2 h of venom administration. Edema was quantified as the percentage increase in weight of the right foot compared to the left foot (negative control). The measurement of the edema percentage is shown in the following formula: [(right foot weight − left foot weight)/left foot weight] × 100.

#### 2.5.4. Myotoxic Activity

Myotoxic activity was determined both in vivo (through the analysis of histological changes in envenomed muscle tissue) and in vitro (by measuring creatine kinase (CK) activity according to the studies [22,23,24]). For each venom (*C. cerastes*, *D. mauritanica* and *B. arietans*), a group of four male mice received 20 μg of the venom dissolved in 100 μL of saline solution into the gastrocnemius muscle. The control group of animals was injected with the same volume of sterile physiological saline solution. Three hours post-injection, the animals were anesthetized, blood samples were collected by cardiac puncture and plasma was obtained by centrifugation [25]. CK activity was determined using a creatine kinase assay kit (Pointe Scientific; Canton, MI, USA) and expressed in international units IU/L. The gastrocnemius muscle was dissected for a histological analysis.

#### 2.5.5. Proteolytic Activity

Casein was used as the substrate to measure the proteolytic activity of the three venoms using the methodology showed in a study by Lomonte and Gutiérrez [26]. Briefly, each venom was tested by incubating 50, 100 and 200 μg of venom protein in 1 mL of casein (1% of casein in phosphate-buffered saline PBS, pH 7.2) in a total volume of 1 mL for 30 min at 37 °C. A negative control was performed without the venom sample. The reaction was stopped by adding 3.0 mL of 5% trichloroacetic acid (Pointe Scientific; Canton, MI, USA). Samples were then centrifuged at 3000× *g* for 10 min at 25 °C. The resulting proteolytic products were evaluated spectrophotometrically at ƛ = 280 nm. The results are presented in units of activity, which is defined as the amount of venom producing an O.D. increase of 1.0 per minute at 280 nm. The specific activity was expressed in units/mg.

### 2.6. Alterations in Skin and Paw

Histological alterations of the skin and paw were observed in mice envenomed by the three venoms *C. cerastes, D. mauritanica* and *B. arietans.* The 20 mice were divided into 5 groups (n = 4 animals per group). Groups 1 and 2 served as skin and paw controls, injected intradermally and via the subplantar route with 100 µL and 50 µL of physiological saline (0.9% NaCl), respectively. Groups 3, 4 and 5 were studied for tissue alterations of the dermal layers of skin and paw and injected with a dose of 0.25 µg/g of the venoms for 2 h.

### 2.7. Neutralizationtoxic Activities

#### 2.7.1. Determination of Median Effective Dose (ED_50_) Neutralization of Venom Lethality

The ability of the commercialized antivenom (*Inoserp-MENA*) to neutralize the lethal effects induced by the three vipers *C. cerastes*, *D. mauritanica* and *B. arietans* was assessed in accordance with the recommendations of the WHO [27,28,29]. This test involves incubation of a fixed dose of venom (“challenge dose”) (3 times the LD_50_) dissolved in saline solution with different amounts of antivenom (250 to 10,000 μg/mouse) at 37 °C for 30 min. Each venom/antivenom mixture was injected intraperitoneally in a group of male Swiss mice (n = 6, 20 ± 2 g). Mice were given free access to food and water, and the survival rate was recorded 48 h after venom injection with monitoring intervals of approximately 3 h. The neutralizing efficacy of the antivenom was expressed as the Median Effective Dose (ED_50_, defined as the amount of reconstituted antivenom that yields 50% survival in venom-exposed animals) or the effective dose ratio (ER_50_, the amount of venom in mg neutralized per ml of antivenom at which 50% of the envenomed mice survived), and the potency of the antivenom (P, the amount of venom completely neutralized by a unit volume of antivenom) [30,31,32,33,34]. The obtained data (ED_50_) were processed through non-linear regression (variable slope, dose–response curve) using GraphPad Prism 7.0 software.

#### 2.7.2. Determination of MHD (Medium Effective Dose) (MHD-ED_50_) for the Neutralization of Hemorrhagic Activity

The in vivo neutralization of hemorrhagic activity induced by the venoms of *C. cerastes*, *D. mauritanica* and *B. arietans* was realized by following the recommendations of the WHO [29]. A challenge dose of venom, equivalent to twice the MHD, was mixed with different doses of commercialized antivenom (*Inoserp-MENA*) and pre-incubated at 37 °C for 30 min. The mixture of venom/antivenom was then intradermally injected into groups of mice (n = 4, 18 ± 2). After 2 h of injection, the skin was removed and the diameter of the hemorrhagic lesion was measured. The control group received venom alone (twice the MHD, positive control). The neutralization capacity was determined as the MHD-median effective dose (MHD-ED_50_), which is defined as the antivenom dose (mg/mL) that reduced a hemorrhagic lesion by 50% when compared to the diameter of the lesion in mice injected with the positive control. The diameter of hemorrhagic lesion was quantified using the method described in the Section 2 (Section 2.5.2).

#### 2.7.3. Determination of Medium Effective Dose (MED-ED_50_) for the Neutralization of Edema-Forming Activity

The efficacy of commercialized antivenom (*Inoserp-MENA*) in neutralizing edema-forming activity caused by the three vipers, *C. cerastes*, *D. mauritanica* and *B. arietans*, was evaluated by pre-incubating a “challenge dose” equivalent to tice the MED of venom with varying amounts of antivenom. After incubation at 37 °C for 30 min, groups of four mice 20 ± 2 g were injected (i.pl) in the right footpad with 50 µL of a mixture containing venom/antivenom, while the left footpad received 50 µL of sterile physiological saline solution alone. Control mice were injected with venom in the right footpad and a saline solution in the left footpad. After two hours, the mice were sacrificed and the percentage of edema forming neutralization was determined as the median effective dose (MED-ED_50_), corresponding to the ratio of venom/antivenom which reduced the oedematic effect induced by the three vipers’ venoms by 50% compared to the control group (venom alone) (taken as 100%). The calculation was evaluated using the method described in the Section 2 (Section 2.5.3).

### 2.8. Neutralization of Myotoxic Activity

The neutralizing ability of commercialized antivenom against myotoxic activity was tested by the preincubation method, by mixing a dose 1 µg/g of each venom with two doses of antivenom, 10 µg/g and 40 µg/g, for 30 min at 37 °C. The mixture was administered via the IM route to a group of mice (n = 4, 18 ± 2). After 3 h, the plasma activity of creatine kinase (CK) released from necrotic muscle fibers was collected by cardiac puncture and assayed. In addition, a histological examination of the muscle tissue samples was performed to corroborate the quantitative results provided by the CK enzymatic assay [24].

### 2.9. Histological Study

Skin, paws and muscles of envenomed mice were dissected and fixed in 10% formaldehyde. After 24 h of fixation, the tissues were dehydrated in ethanol, cleared in xylene, impregnated and embedded in paraffin wax. Then, transverse sections of 4 µm in thickness were prepared and stained with Hematoxylin and Eosin for histopathological examinations [35,36,37].

### 2.10. Statistical Analysis

The tests were performed at least three times and data are represented as means ± standard deviations (SD). Significant differences between all experimental tests were analyzed by a one-way ANOVA test using GraphPad Prism 7.0 software. Significance was accepted at *p* > 0.05 (ns), *p* < 0.05 (*), *p* < 0.01 (**), *p* < 0.001 (***) and *p* < 0.0001 (****). The LD_50_ of the venoms and ED_50_ of antivenoms were calculated by non-linear regression (variable slope) using GraphPad Prism 7.0 software. For comparative purposes, ED_50_ values of antivenoms were normalized (n-ED_50_) by their respective protein amount and expressed as milligrams of venom neutralized per gram of antivenom protein (mg/g).

## 3. Results

### 3.1. Median Lethal Dose (LD_50_)

**Figure 2:**
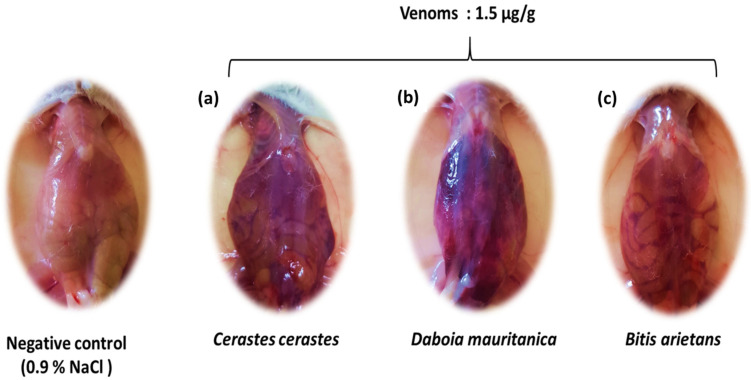
The hemorrhagic effect induced by the venoms of the vipers *Cerastes cerastes* (**a**), *Daboia mauritanica* (**b**) and *Bitis arietans* (**c**) which were injected with the same amount (1.5 µg/g) intraperitoneally. After 24 h, the mice were sacrificed and photos were taken immediately.

The median lethal dose (LD_50_) of the venom of the three vipers *C. cerastes*, *D. mauritanica* and *B. arietans* is 36.30 µg/mouse (95% confidence interval: 30.98–40.57) (1.815 (1.549–2.028) µg/g), 48.64 µg/mouse (95% confidence interval: 47.12–49.86) (2.432 (2.356–2.493) µg/g) and 78.06 µg/mouse (95% confidence *interval*: 70.87–82.89) (3.903 (3.543–4.145) µg/g), respectively (Table 1). The results showed that *C. cerastes* venom is more toxic than both *D. mauritanica* and *B. arietans* venom. A toxicity assessment was performed for the three venoms and hemorrhagic syndrome was observed in the examination of the peritoneal cavity of mice 24 h post-venom injection compared to the control group (Figure 2).

### 3.2. Electrophoretic Profiling

**Figure 3:**
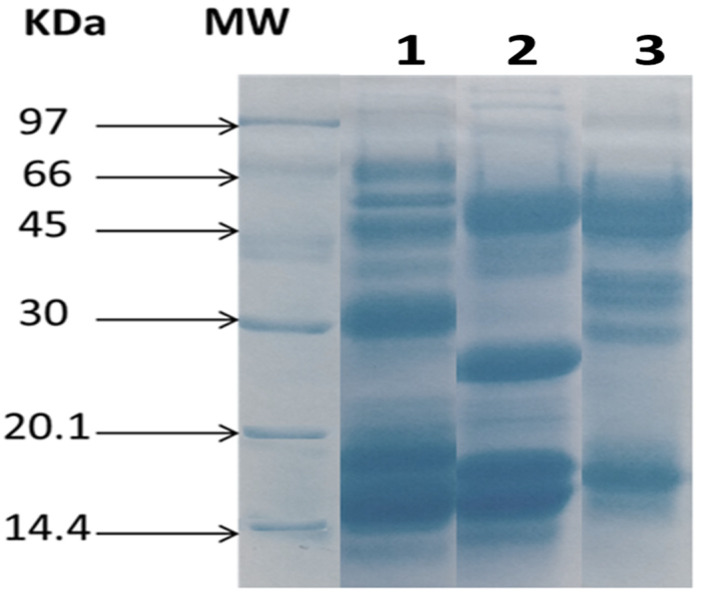
SDS-PAGE profiles of venoms. Electrophoretic separation of venoms was performed on a vertical slab of 12% acrylamide under reducing conditions. MW: molecular mass markers; lane 1: *B. arietans* venom; lane 2: *D. mauritanica* venom; lane 3: *C. cerastes* venom. The markers indicated on the left are expressed as kDa. Gel was stained using Coomassie blue.

The results of electrophoresis, based on the separation of proteins according to molecular weight, show that the venoms of *C. cerastes*, *D. mauritanica* and *B. arietans* differ in composition and generally contain high molecular weight (MW) proteins. The *C. cerastes* venom revealed seven bands, of which three had a higher intensity than others, with a molecular weight ranging from 40 to 55 kDa, while that of *D. mauritanica* presented nine bands, including four of high intensity with molecular masses ranging from 20 kda to 60 kDa. The *B. arietans* venom revealed a complex electrophoretic profile with the presence of twelve bands, of which five are intense (Figure 3).

### 3.3. Proteolytic Activity

The results obtained showed the ability of the *C. cerastes*, *D. mauritanica* and *B. arietans* venom to hydrolyze casein, thus revealing a proteolytic activity. According to the obtained results, the proteolytic activity of *C. cerastes* venom is more than the other viper’s venom, which were estimated at 67.43 ± 2.8 U/mg, 61.8 ± 3.04 U/mg and 44 ± 1.23 U/mg for the *C. cerastes*, *D. mauritanica* and *B. arietans* venoms, respectively (Table 1).

### 3.4. Hemorrhagic Activity

**Figure 4:**
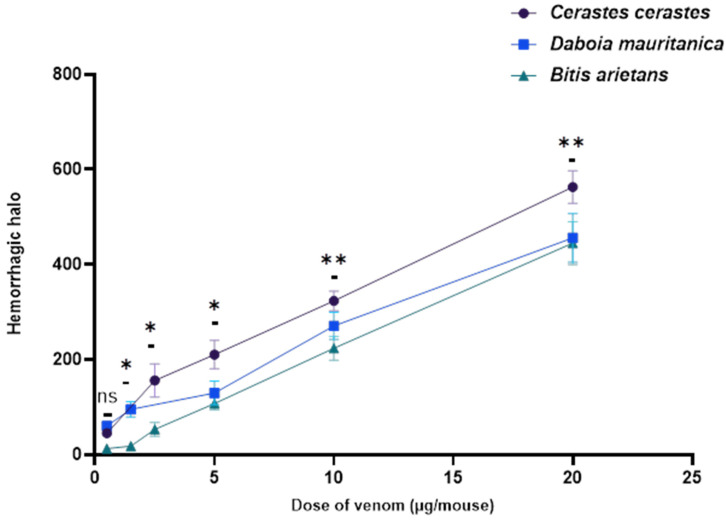
Dose–response curve of hemorrhagic activity induced by different concentrations of *Cerastes cerastes*, *Daboia mauritanica* and *Bitis arietans* venom in mice. The graph represents the mean of hemorrhagic halos calculated using graph paper ± SD (standard deviation) (n = 4). The linear regression of hemorrhagic halos according to venom dose is shown (r = 0.9847 for *C. cerastes*, r = 0.9914 for *D. mauritanica* and r = 0.9988 for *B. arietans*). The data were analyzed using a one-way ANOVA to evaluate statistical significance: ** (*p* < 0.01), * (*p* < 0.05) and ns (not significant) (*p* > 0.05). The symbol ‘*’ specifically indicates significance compared to the control group (physiological solution).

The minimal hemorrhagic dose (MHD) was used to evaluate the hemorrhagic activity. Macroscopic observations of the inner surface of mice skin removed 2 h after intradermal injection of *C. cerastes* and *D. mauritanica* venom showed significantly more hemorrhagic areas than after injection with *B. arietans* venom (Figure 7). The study’s findings revealed that the venoms of the three viper species were able to induce strong hemorrhages in a dose-dependent manner. Notably, the venom of *C. cerastes* exhibited the most potent hemorrhagic effects, with an MHD of 0.34 ± 0.11 μg, which was lower than that of the other two viper species. In contrast, the MHDs of *D. mauritanica* and *B. arietans* were approximately 1.37 ± 0.89 μg and 3.70 ± 1.88 μg, respectively (Figure 4).

### 3.5. Edema-Forming Activity

**Figure 5:**
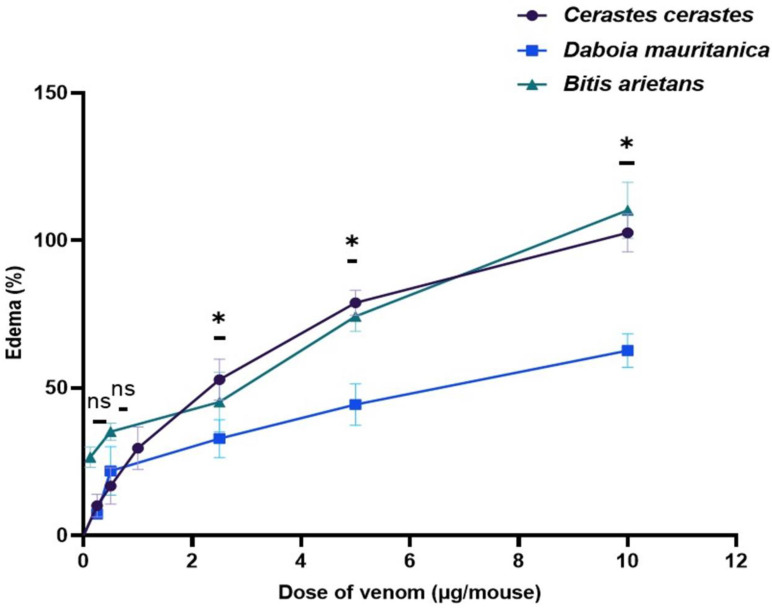
Dose–response curve of edema-inducing activity induced by different concentrations of *Cerastes cerastes*, *Daboia mauritanica* and *Bitis arietans* venom in mice. The graph expresses the percentage of the weight of the venom-injected footpads compared to the weight of the saline-injected footpads. Values represent the means ± SD (standard deviation) (n = 4). The linear regression of the doses of venom according to the percentage of edema (%) is indicated (r = 0.8946 for *C. cerastes*, r = 0.9144 for *D. mauritanica* and r = 0.9845 for *B. arietans*). The data were analyzed a one-way ANOVA to evaluate statistical significance: * (*p* < 0.05) and ns (not significant) (*p* > 0.05). The symbol ‘*’ specifically indicates significance compared to the control group (physiological solution).

The minimum edema-forming dose (MED) was used to evaluate the formation of edema. Macroscopic observations 2 h post-sub-plantar venom injection showed significant edema hemorrhages at the injection site of the footpad (Figure 8). The results obtained showed that the venoms of the three vipers are able to induce strong edema activity with a dose-dependent effect. *B. arietans* venom (MED = 0.23 ± 0.1 μg) has a higher edema-forming activity compared to *C. cerastes* venom with an MED of 1.74 ± 0.82 μg, while *D. mauritanica* venom showed a lower edema activity than the other vipers (MED = 3.05 ± 1.30 μg) (Figure 5).

### 3.6. Myotoxic Activity

**Figure 6:**
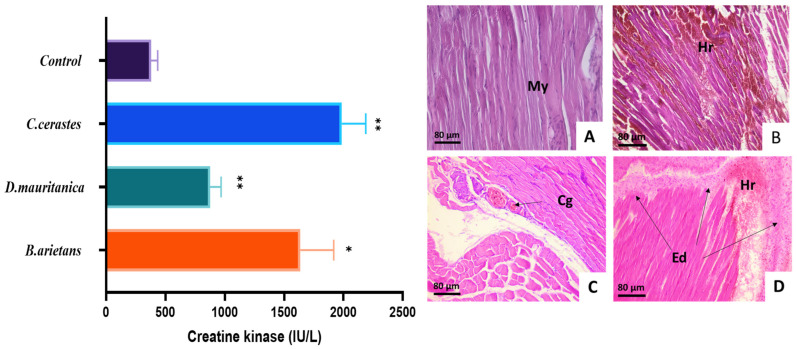
Histological analysis and myotoxicity induced by *C. cerastes, D. mauritanica* and *B. arietans* venom in mouse gastrocnemius muscle. After 3 h of injection, the plasma samples were collected for quantification of the creatine kinase activity (CK) by cardiac puncture and muscle tissues were drawn by histological analyses. Histological sections in the gastrocnemius muscle of mice envenomed with a dose (1 µg/g) of *Cerastes cerastes* (**B**), *Daboia mauritanica* (**C**) and *Bitis arietans* (**D**) venom. (Control, (**A**)) Mice received 0.9% NaCl solution. My: Myofibril, Hr: Hemorrhage, Ed: Edema, Cg: Congestion of the blood vessels. The CK activity was quantified using a commercial kit. Results are expressed as means ± S.D (n = 4) and were analyzed using a one-way ANOVA test, ** *p* < 0.01, * *p* < 0.05 and ns (not significant) > 0.05. ‘*’ indicates significance compared to the control group (physiological solution).

Pathological alterations were observed in striated muscle tissue samples from mice injected with 1 µg/g of the venom of *C. cerastes*, *D. mauritanica* and *B. arietans* over a period of 3 h (Figure 6). Tissue sections from control mice injected with saline solution showed normal histological features of skeletal muscle tissue **(**Figure 6A). Muscle fiber sections from mice injected with *C. cerastes* (Figure 6A) and *D. mauritanica* (Figure 6C) venom showed intense hemorrhages with red blood cell extravasation, abundant edema in the interstitial space and vascular congestion. Additionally, a polymorphic inflammatory infiltrate composed of lymphocytes, neutrophils and macrophages was also observed. The group of mice injected with *B. arietans* venom had more hemorrhages and edemas than those injected with the other two vipers’ venom, and significant necrosis of skeletal muscle fibers was present. Furthermore, there were inflammatory infiltrates rich in neutrophils and lymphocytic nodules (Figure 6D). On the other hand, all the venoms increased the plasma CK levels, which were collected 3 h after intramuscular injection (Figure 6). Compared to the corresponding values in the serum of control mice (379.667 ± 55.33 IU/L), the CK values of the venoms *C. cerastes* (1986.4 ± 201.18 IU/L), *D. mauritanica* (876.66 ± 92.9 IU/L) and *B. arietans* (1636.67 ± 281.12 IU/L) were significantly higher, with *C. cerastes* venom causing the greatest increase in CK compared to the other venoms. These findings are consistent with the muscle damage observed in the histological analysis, which occurs soon after envenomation.

### 3.7. Histopathological Analysis

Histopathological alterations were studied in the paws, skin and muscle of mice. The observed alterations were compared to those observed in control mice which were injected with physiological solution and showed normal histological features in the skin (Figure 8) and paws (Figure 9).

#### 3.7.1. Skin Alterations

**Figure 7:**
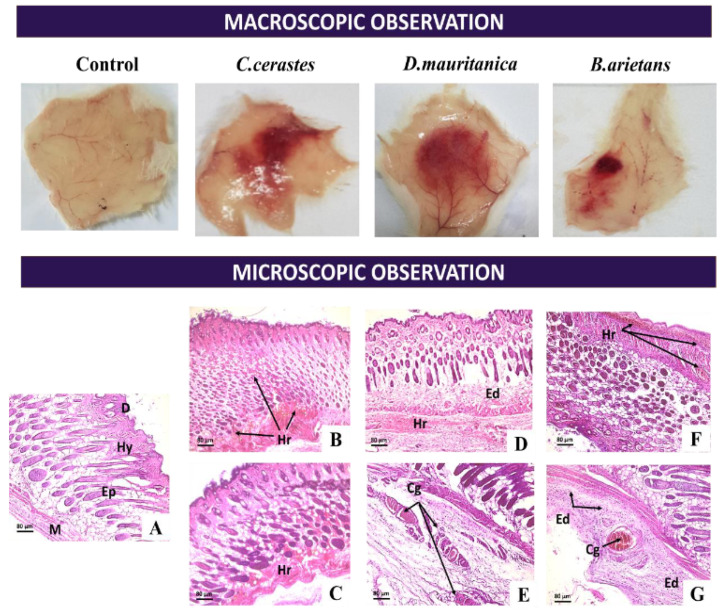
Macroscopic and microscopic observations of the dorsal surface of mice 2 h after intradermal injection of venom (0.25 µg/g). Skin lesions generated after intradermal injection of 0.25 µg/g of *C. cerastes* (**B**,**C**), *D. mauritanica* (**D**,**E**) and *B. arietans* (**F**,**G**) venom in mice. The control group was injected with 100 µL of saline solution (**A**). D: Dermis, Ep: Epidermis, Hy: Hypodermis, Mu: Muscle, Hr: Hemorrhage, Ed: Edema, Cg: Congestion of vessels, If: Mononuclear inflammatory infiltrate.

Histological examinations of skin tissue in mice injected with a fixed dose (0.25 µg/g) of the venom of *C. cerastes* (Figure 7B,C), *D. mauritanica* (Figure 7D,E) and *B. arietans* (Figure 7F,G) showed significant lesions from the subepidermal dermis to the hypodermis. They consisted of edemas, which were slight or moderate, and superficial and/or deep hemorrhages, depending on the venom injected. We also noticed a degeneration of collagen fibers with an infiltration by inflammatory cells. The injection of *C. cerastes* venom resulted in a significant hemorrhage in the hypodermis, associated with congestion of dilated vessels. The dermis was slightly edematous. On the other hand, *D. mauritanica* venom caused deeper hemorrhages, extending to the sub-hypodermal muscle. Elsewhere, lesions similar to those caused by *C. cerastes* venom were observed. *B. arietans* venom showed different results compared to *C. cerastes* and *D. mauritanica* venom at the cutaneous level, showing superficial hemorrhages and moderate to deep edemas with the presence of minimal, scattered and essentially mononuclear inflammatory infiltrates.

#### 3.7.2. Paw Alterations

**Figure 8:**
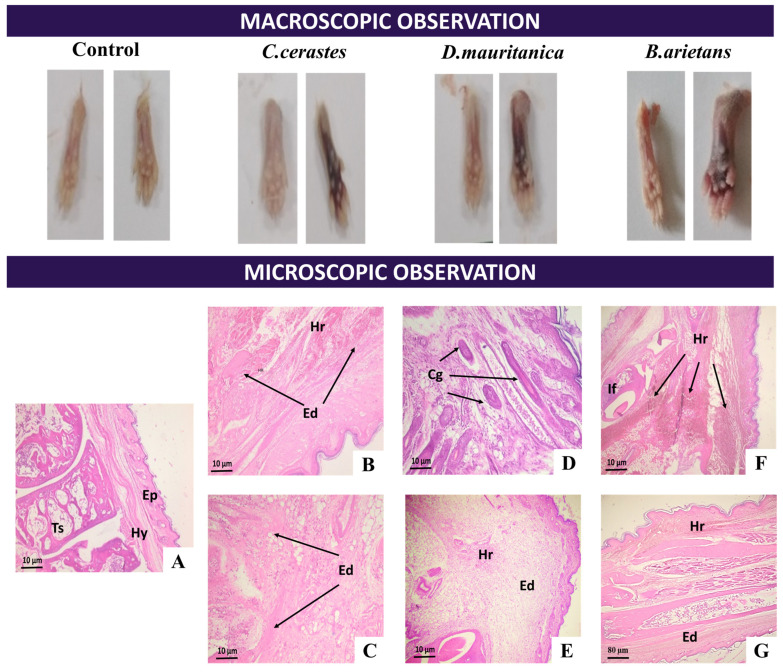
Macroscopic and microscopic observations of mouse paws 2 h after subplantar injection of venom (0.25 µg/g). Histological sections of the *C. cerastes* (**B**,**C**), *D. mauritanica* (**D**,**E**) and *B. arietans* (**F**,**G**) venom in mice. The control group was injected with 100 µL of saline solution (**A**). Ep: Epidermis, Hy: Hypodermis, Ts: Osteo-medullary tissue, Hr: Hemorrhage, Ed: Edema, Cg: Congestion of the vessels, If: Mononuclear inflammatory infiltrate.

After subplantar injection of *C. cerastes* (Figure 8B,C), *D. mauritanica* (Figure 8D,E) and *B. arietans* (Figure 8F,G) venom, sections of the paw revealed edema at the injection site, with the presence of hemorrhages and inflammation located mainly in the dermal and muscle layers with increasing doses of venoms. A dose of 0.25 µg/g induced intense edema, characterized by discontinuous detachment of the epidermis from the dermis, a hemorrhagic area associated with a severe congestion of vessels and minimal mononuclear inflammatory infiltrate. *B. arietans* venom is responsible for the most severe pathological changes in the paws of mice.

### 3.8. Neutralization Capacity

#### 3.8.1. Neutralization of Lethality

In this part of the study, we tested the efficacy of the antivenom *Inoserp-MENA*, which is the only polyvalent commercialized antivenom in Morocco. The highest neutralizing potency was observed in the test against *C. cerastes* venom (1.51 mg/mL), with an ED_50_ of 38.13 µL (95% CI: 32.74–44.19). This suggests that the antivenom could potentially neutralize the lethal effects of the venom of this species. Alarmingly, the antivenom showed a higher ED_50_ value of 201 µL (95% CI: 1.8078–214.5 μg/mL) for *D. mauritanica* venom, indicating a lower efficacy than that for *C. cerastes* venom. Unfortunately, the antivenom could not neutralize the lethal effect of *B. arietans* venom. The mice died at a maximum dose of 400 µL of antivenom; four out of five injected mice died. Table 2 presents these results.

#### 3.8.2. Neutralization of Hemorrhagic and Edema-Forming Activity

Table 3 demonstrates the efficacy of the commercialized antivenom (*Inoserp-MENA*) in neutralizing the hemorrhagic and edematic effects of the three venoms, measuring the median effective dose (ED_50_) in μL and mg/mL (95% ci) and the normalized median effective doses (n-ED_50_) in mg/g. The antivenom was found to be more effective in countering the hemorrhagic effect of *C. cerastes* venom than *D. mauritanica* venom. However, a higher dose of *Inoserp-MENA* antivenom was required to neutralize the hemorrhagic effect induced by *B. arietans* venom compared to the other venoms.

The ability to neutralize edema formation is lower for both *C. cerastes* and *D. mauritanica*. In the case of *B. arietans*, the protection against edema formation is almost negligible, with a six times lower potency than both venoms.

#### 3.8.3. Neutralization In Vitro of Myotoxic Activity

**Figure 9:**
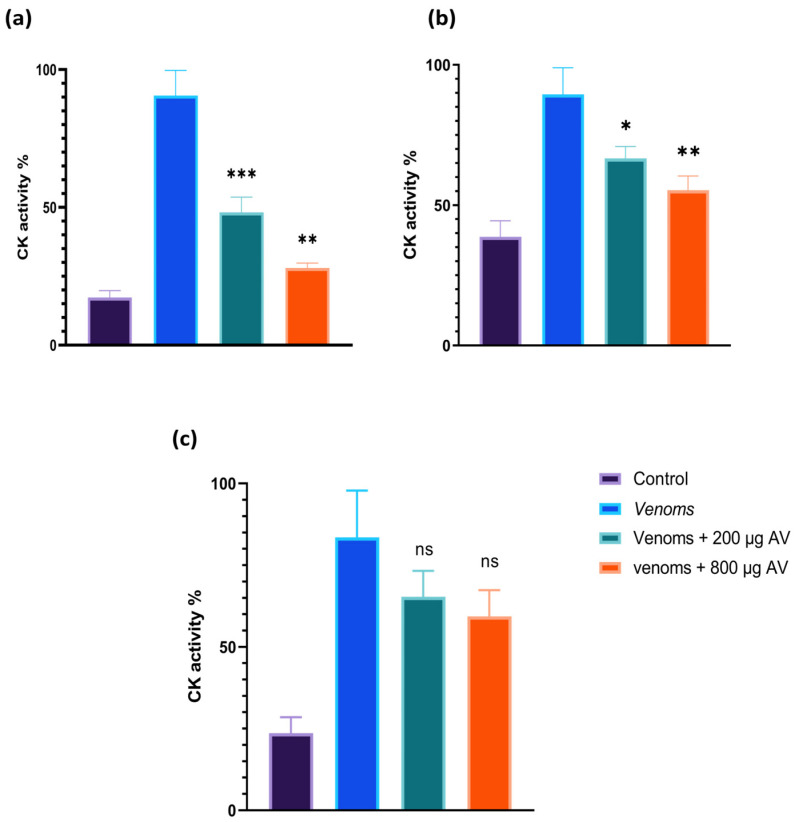
Neutralization of *C. cerastes* (**a**), *D. mauritanica* (**b**) and *B. arietans* (**c**) venom-induced myotoxicity by commercialized antivenom *Inoserp-MENA* using the murine model. The study involved mixing two doses of antivenom with 20 µg of venom, followed by a 30 min incubation at 37 °C. The mixture was then injected intramuscularly in mice, and the plasma creatine kinase (CK) activity was determined after 2 h. Each point represents the mean ± SD, analyzed using a one-way ANOVA test. *** *p* < 0.001, ** *p* < 0.01, * *p* < 0.05 and ns (not significant) > 0.05. ‘*’ indicates significance compared to the envenomed groups (negative control).

The administration of a dose of *Inoserp-MENA* antivenom significantly reduced *C. cerastes* venom-induced myonecrosis, as assessed by the CK activity levels in the plasma. Nearly 20% neutralization was observed with a high quantity of antivenom compared to the group of envenomed mice. Furthermore, the same amount of antivenom was able to block 50% of the myotoxic activity induced by *D. mauritanica* venom. However, the antivenom demonstrated weak protection against the myotoxic activity of *B. arietans* venom and lower stabilization of the creatine kinase levels compared to the other two venoms (Figure 9).

## 4. Discussion

Envenomation by vipers is the most dangerous form of snakebite in Morocco. This study focuses on the adverse effects caused by the most medically important vipers, namely *Daboia mauritanica* (45.5% of cases), *Cerastes cerastes* (10.9% of cases) and *Bitis arietans* (10.9% of cases). The main objective of this study is to compare the toxic activities of the venom from these vipers to better understand the different physiopathological effects induced by viper envenomation. Additionally, the study examines the neutralizing power of a commercialized antivenom (*Inoserp-MENA*) for these effects, thus providing data for optimal management of viper envenomation victims.

In this study, the LD_50_ values showed that the toxicity of the *C. cerastes* venom is greater than that of *D. mauritanica*, while *B. arietans* venom has almost half the toxicity of *C. cerastes*. Our results of the LD_50_ of *C. cerastes* venom were similar to other studies previously conducted on North African horned vipers. For example, a study by Karam and Mohamed [38] showed an LD_50_ of 1.09 µg/g for the venom of the Egyptian *Cerastes cerastes* after injection intraperitoneally in male Swiss mice (weight 20 ± 2 g), while the LD_50_ of *Cerastes cerastes* venom from Algeria was established at 1.5 µg/g in male NMRI mice (20 ± 2 g body mass) by injecting the venom intraperitoneally [39,40]. Concerning *D. mauritanica* venom, Makran and coworkers [41] determined an LD_50_ of 1.856 ± 0.93 µg/g using the same injection route and the same type and weight of mice, while its Algerian cousin showed a lower toxicity, estimated at 2.5 µg/g (i.p injection, male NMRI mice) [42]. However, for *B. arietans*, which is considered among the most fatal African snakes, a study reported by Sánchez and coworkers [43] indicated that intravenous injection of *Bitis arietans* venom from Nigeria and Cameroon revealed LD_50_ values of 0.68 µg/g (0.58–0.77) and 1.12 µg/g (0.95–1.53), respectively. Furthermore, Oukkache’s study [44] showed the toxic activity of the venom of Moroccan vipers *D. mauritanica*, *C. cerastes* and *B. arietans*, matching our results, since the LD_50_ (i.p injection, male Swiss mice) of *B. arietans* venom (1.627 μg/g) was higher than that of *C. cerastes* (0.3788 μg/g) and *D. mauritanica* (0.29 μg/g). All these results prove that the intraspecific variation in snake venom is a ubiquitous phenomenon, particularly evident in species with a wide range of distribution, especially for the *C. cerastes* viper, which is more widespread compared to *D. mauritanica* and *B. arietans* in North Africa [45,46,47]. Of note, several factors such as sex, age, diet, genetic strain and animal health seem to be involved.

The electrophoretic profile of the three crude venoms of *C. cerastes*, *D. mauritanica* and *B. arietans* revealed proteins with different molecular masses ranging from 14.4 to 70 kDa. The protein composition of each venom is heterogeneous, showing several protein bands of varying intensity. *C. cerastes* venom revealed seven bands, of which three have a higher intensity than the others, with a molecular weight ranging from 40 to 55 kDa, while that of *D. mauritanica* presented nine bands, including four of high intensity, with molecular masses ranging from 20 kda to 60 kDa. Both *C. cerastes* and *D. mauritanica* venom have bands with almost the same molecular masses, giving us a general idea of the main protein families found in these snake venoms, especially the metalloproteinases (SVMPs), which explains their strong hemorrhagic and proteolytic activities. These results are similar to those of a proteomic study of *C. cerastes* and *D. mauritanica* venom showing the presence of 25 toxins belonging to six protein families, mainly class PIII metalloproteinases (55.3%) and phospholipases A_2_ (19.1%) for *C. cerastes*, and 45 toxins belonging to nine protein families with molecular masses ranging from 14 to 70 kDa and mainly comprising type III metalloproteinases (PIII-SVMPs) (34%) and disintegrins (13.8%) for *D. mauritanica* [41,48]. *B. arietans* venom revealed a complex electrophoretic profile, with the presence of 12 bands of which 5 are intense. This matches with several studies which presented a complex proteome of venoms of the genus *Bitis*, including toxins from between 11 an 14 enzyme families, in particular SVSP, SVMP and PLA_2_ [49,50].

The study of proteolytic activity was also studied using casein as a substrate. This activity was evaluated at 67.43 ± 2.8 U/mg, 61.8 ± 3.04 U/mg and 44 ± 1.23 for *C. cerastes*, *D. mauritanica* and *B. arietans* venom, respectively. These values are in agreement with the results of Boumaiza et al., 2016, who showed that the *C. cerastes* venom of Algeria has a higher caseinolytic activity than that of *D. mauritanica*. On the other hand, *C. cerastes* venom of Morocco had a low proteolytic activity compared to that of *D. mauritanica* [44], while the study of Markan [41] showed the identical activities of both *C. cerastes* and *D. mauritanica* venom. On the other hand, there is no study characterizing the proteolytic activity of *B. arietans* venom from Morocco [47], but the venom of *Ba* from different African countries (Nigeria, Ghana, Malawi and Tanzania) was found to degrade the substrates of fibrinogen and gelatin. This variation between the three species is due to the concentration of certain proteases (SVMP and SVSP) which recognize the substrate (casein, gelatin and fibrinolysis), as reported by these studies [51,52].

Some authors have also hypothesized a possible correlation between the proteolytic activity of snake venom and their hemorrhagic activity [53,54]. *C. cerastes* and *D. mairitanica* venom exhibited MHDs of 0.34 ± 0.11 μg and 1.37 ± 0.89 μg, respectively. On the other hand, *B. arietans* venom revealed an MHD of 3.70 ± 1.88 µg, which indicates a weak hemorrhagic activity compared to *C. cerastes* and *D. mauritanica* venom. Our results are similar to numerous studies showing the hemorrhagic effect in mice (18–22 g) by intradermal injection of various venoms of North African vipers [55,56], which reported MHDs ranging from 0.13 µg to 22.34 µg for *Cerastes cerastes* venom and hemorrhagic activities ranging from 0.63 to 19.39 µg for *Daboia mauritanica* venom [38,40,44,57]. Sanchez and coworkers [43] studied the biological effects of *Ba* venom from Cameroon and Nigeria, such as hemorrhagic activity, showing MHDs of 0.24 ± 0.03 and 0.625 ± 0.01 µg, respectively, 2 h post-intradermal injection of Swiss mice with a body weight of 20 ± 2 g. On the other hand, there have been no studies on Moroccan *B. arietans*. Numerous studies, including ours, have demonstrated that the hemorrhagic activity of pit vipers is associated with skin tissue damage [58,59,60,61]. The abundance of PIII-SVMPs in *C. cerastes* (57.3%) and *D. mauritanica* (34%) venom and their severe hemorrhagic activity indicates the involvement of class P-III SVMPs in the pathogenesis of local tissue damage by the hydrolysis of the key substrates of basement membrane microvessels such as laminin, type IV collagen and nidogen/entactin [48,62,63,64].

Besides the local damage caused by these venoms, we also have the induction of edema. Our results demonstrate that intraplantar injection of increasing doses of three vipers’ venom in mice induced a significant increase in vascular permeability, followed by edema in the injected paws. *B. arietans* venom exhibited a high edematous activity followed by *C. cerastes* and *D. mauritanica* venom, with MDEs of 0.23 ± 0.1 μg, 1.74 ± 0.82 µg and 3.05 ± 1.30 μg, respectively. Edema is caused by most pit viper venoms at different degrees depending on the species. For example, the venom of the *Bothrops* species, e.g., *B. jararaca*, *B. jararacussu*, *B. neuwiedii* and *B. alternatus*, induced greater hypertrophy of the paws of laboratory animals (>70%) [65]. In agreement, *C. cerastes* venom revealed an edema-forming activity similar to that of its Algerian congener (DME of 1.4 µg) [39]. In contrast to our results, Makran’s study [41] reported that *D. mauritanica* venom is more edematic than that of *C. cerastesi*, with MDE values 3.84 ± 4.03 and 1.02 ± 1.36 µg, respectively. An increased vascular permeability is the main mechanism of edema formation and depends on the production and/or release of inflammatory mediators at the site of injury, mainly histamine, eicosanoid and bradykinin [65]. Nevertheless, other studies have implicated LAAOs isolated from *Viperidae* and *Elapidae* venom in different pathogenetic aspects of envenomation, notably edema [66]. We can therefore conclude that the higher edema forming ability of *B. arietans* venom compared to *C. cerastes* and *D. mauritanica* venom is probably related to the collective action of PLA_2_ and LAAO enzymes, since the proteomes of *C. cerastes* and *D. mauritanica* venom do not contain LAAO, unlike the venom proteome of Nigerian *B. arietans*, which contains 8.72% LAAO and 10% PLA_2_ [48,48,50].

In addition to edema, PLA_2_ is also responsible for muscle damage by disrupting the integrity of the plasma membrane of the skeletal muscle [67,68]. In addition to PLA_2_, viper venom contains hemorrhagic SVMPs, which contribute to pathogenesis in the muscle tissue, generating ischemia [67]. In the case of *Viperidae* venom, such as the genus *Cerastes, Daboia* and *Bitis*, which contain myotoxic and hemorrhagic enzymes, myonecrosis mainly occurs via a combination of the direct action of myotoxins on muscle fibers, as well as indirectly, by ischemia resulting from venom-induced vascular damage. Interestingly, the *B. arietans* venom group caused significantly more hemorrhage and edema events in comparison to other venoms. These results suggest the strong presence of PLA_2_ in the proteome of *B. arietans* venom, since there are no studies reported yet on the biological, enzymatic and proteomic activities of Moroccan *B. arietans* venom.

Although the species examined in this study have demonstrated lethal incidence and pathological variation, the only treatment available to counteract Moroccan viper venom is the polyvalent antivenom *Inoserp-MENA.* We conducted a study to evaluate the efficacy of this antivenom serum in vivo by testing its ability to neutralize the toxic and lethal activities of the venom of *C. cerastes*, *D. mauritanica* and *B. arietans*, following the guidelines set forth by the World Health Organization (WHO) [29]. According to our findings, *Inoserp-MENA* effectively neutralized the toxic activity and pathological properties, such as the hemorrhagic and edematous activity and myotoxicity induced by *C. cerastes* venom. However, for protection against the same effects caused by *D. mauritanica* venom, a higher dose of antivenom was required, almost twice as high as that for the venom of *C. cerastes*. Unfortunately, *Inoserp-MENA* was unable to neutralize the toxic and edema-forming activity of *B. arietans* venom, but provided a low protection against hemorrhagic and myotoxic activity. This suggests that the antivenom used does not exhibit complete immune reactivity against *B. arietans* venom. The partial neutralization of the hemorrhagic and myotoxic activities can be explained by cross-reactivity with venom from other snake species. Venom from different snake species may contain similar or partially similar components, which can lead to some degree of neutralization of the hemorrhagic activity, albeit less effective than against the toxins specific to *B. arietans* venom. Our study aligns perfectly with a recent investigation into the effectiveness of *Inoserp-MENA* antivenom against the *C. cerastes* viper and the *Cerastes* genus as a whole [68]. The limited efficacy of the antivenom against the venom of *D. mauritanica* and *B. arietans* can be explained by the findings from a study by Alomran [69]. This study presented experimental evidence indicating that incorporating multiple venoms into an immunogen mixture may have adverse effects on effectiveness. This effect could arise from the potential dilution of venom-neutralizing antibodies that are specific to a particular venom due to the inclusion of numerous immunogens, including toxins specific to additional venoms used for vaccination [56,70,71,72]. Multiple studies have demonstrated the limited ability of commercialized antivenoms to neutralize venom, highlighting the pressing need to develop effective antivenom sera. Achieving this requires a systematic understanding of venom variability, as well as local production targeted toward the species which the antivenom will be used against. These measures can significantly improve the strategy of antivenom production and save the lives of millions of snakebite victims in Morocco.

## 5. Conclusions

In the present study, *B. arietans* venom analyses were carried out for the first time in Morocco and compared to *C. cerastes* and *D. mauritanica*. We calculated experimentally the LD_50_ values and pathophysiological effects of three types of viper venom in mice. The venom of *C. cerastes* and *D. mauritanica* are toxic, showing substantial hemorrhagic activities, whereas the venom of *B. arietans* causes intense edema. As the presently available antivenom in Morocco seems to be ineffective (for *D. mauritanica* and *B. arietans* venom), the LD_50_ values and pathophysiological results obtained in this study can be employed to create powerful antivenom sera for Moroccan species.

## Figures and Tables

**Table 1:** Biologic and enzymatic activities of the three Moroccan viper’s venom.

Venom	Lethality Potency		Hemorrhagic Activity	Edematogenic Activity	Myotoxic Activity	Proteolytic Activity
	LD_50_ (µg/mouse) ^a^	LD_50_ (µg/g)	MHD (μg) ^b^	MED (μg) ^c^	(IU/L)	U/mg ^d^
*Cerastes cerastes*	36.30 (30.98–40.57)	1.815 (1.549–2.028)	0.34 ± 0.11	1.74 ± 0.82	1986.4 ± 201.18	67.43 ± 2.8
*Daboia mauritanica*	48.64 (47.12–49.86)	2.432 (2.356–2.493)	1.37 ± 0.89	3.05 ± 1.30	876.6 ± 92.91	61.8 ± 3.04
*Bitis arietans*	78.06 (70.87–82.89)	3.903 (3.543–4.145)	3.70 ± 1.88	0.23 ± 0.1	1636.67 ± 281.12	44 ± 1.23

^a^ Median lethal dose in μg/mouse. The 95% confidence interval is in parentheses. It was obtained by the study of the dose–response curve of mice injected intraperitoneally (i.p.) with different amounts of venom; ^b^ Minimal hemorrhagic dose in μg ± standard deviation; ^c^ Minimum edema-forming dose in μg ± standard deviation; ^d^ Units of caseinolytic activity per milligram of venom ± standard deviation.

**Table 2:** Neutralization of the lethality of the venom of *C. cerastes*, *D. mauritanica* and *B. arietans* using commercial antivenom (*Inoserp-MENA*).

Venoms	i.p. LD_50_ (μg/g)	Challenge Dose (μg/g)	ED_50_ ^a^ (μL of Antivenom)	ER_50_ ^b^ (mg/mL)	P (mg/mL) ^c^	Normalized P (mg/g) ^d^
*Cerastes cerastes*	1.815 (1.549–2.028)	3 LD_50_	38.13 (32.74–44.19)	2.89	1.93	95.20
*Daboia mauritanica*	2.432 (2.356–2.493)	3 LD_50_	201 (1.8078–214.5)	0.72	0.486	24.1
*Bitis arietans*	3.903 (3.543–4.145)	3 LD_50_	>400	-	-	-

^a^ ED_50_: Median effective dose, dose of antivenom (µL) at which 50% of mice survived. The 95% confidence interval is in parentheses. Concentration of antivenom is 20 mg/mL; ^b^ ER_50_ (median effective ratio), ratio of venom (mg) to the volume does of antivenom (mL) at which 50% of mice survived (95% c.i.); ^c^ P (potency), amount of venom (mg) completely neutralized per unit volume of antivenom (mL); ^d^ n-P (normalized potency), the neutralization potency of antivenom at which the amount of venom (mg) completely neutralized per unit amount of antivenom protein (g).

**Table 3:** Neutralization of hemorrhagic and edema-forming activities of the venoms *C. cerastes*, *D. mauritanica* and *B. arietans* using commercial antivenom (*Inoserp-MENA*).

	**Neutralization of Hemorrhagic Activity**		
	**ED_50_ ^a^ (μL)**	**ED_50_ ^b^ (mg/mL )**	**n-DE_50_ ^c^ (mg/g)**
*Cerastes cerastes*	3.644 (3.193–4.179)	0.18 (0.21–0.16)	18.66
*Daboia mauritanica*	18.90 (16.62–21.52)	0.14 (0.16–0.12)	14.50
*Bitis arietans*	68.21 (60.81 to 77.11)	0.10 (0.12–0.09)	10.84
	**Neutralization of Edema-Forming Activity**		
	**ED_50_^a^ (μL)**	**ED_50_^b^ (mg/mL)**	**n-DE_50_^c^ (mg/g)**
*Cerastes cerastes*	21.98 (18.99–25.42)	0.16 (0.18–0.13)	15.83
*Daboia mauritanica*	16.67 (14.41–19.28)	0.35 (0.42–0.311)	36
*Bitis arietans*	18.02 (14.94–21.71)	0.0255 (0.0307–0.0211)	2.55

The challenge dose used was twice the MHD and MED values. The concentration of antivenom was 10 mg/mL; ^a^ ED_50_: the median effective dose was defined as the dose of antivenom (µL) at which the venom’s hemorrhagic and edema-forming activity was reduced by 50%. The 95% confidence interval is in parentheses. ^b^ Median effective dose, the ratio was defined as the amount of venom neutralized per unit volume of antivenom (mg venom/mL antivenom) at which the venom’s hemorrhagic activity was reduced by 50% (95% c.i.). ^c^ n-ED_50_ normalized median effective dose, the median effective dose of antivenom at which the amount of venom (mg) is neutralized per unit amount of antivenom protein (g).

## Data Availability

Not applicable.
